# A role for calcium in resistin transcriptional activation in diabetic hearts

**DOI:** 10.1038/s41598-018-34112-4

**Published:** 2018-10-23

**Authors:** Rajvir Singh, Pedro Moreno, Roger J. Hajjar, Djamel Lebeche

**Affiliations:** 1 Cardiovascular Research Institute, Department of Medicine, Icahn School of Medicine at Mount Sinai, New York, New York, 10029 USA; 2 Diabetes, Obesity and Metabolism Institute, Department of Medicine, Icahn School of Medicine at Mount Sinai, New York, New York, 10029 USA; 3 Graduate School of Biological Sciences, Department of Medicine, Icahn School of Medicine at Mount Sinai, New York, New York, 10029 USA

## Abstract

The adipokine resistin has been proposed to link obesity, insulin resistance and diabetes. We have previously reported that diabetic hearts express high levels of resistin while overexpression of resistin in adult rat hearts gives rise to a phenotype resembling diabetic cardiomyopathy. The transcriptional regulation of resistin in diabetic cardiac tissue is currently unknown. This study investigated the mechanism of resistin upregulation and the role of Serca2a in its transcriptional suppression. We demonstrate that restoration of Ca^2+^ homeostasis in diabetic hearts, through normalization of Serca2a function genetically and pharmacologically, suppressed resistin expression via inhibition of NFATc. H9c2 myocytes stimulated with high-glucose concentration or Ca^2+^ time-dependently increased NFATc and resistin expression while addition of the Ca^2+^ chelator BAPTA-AM attenuated this effect. NFATc expression was enhanced in hearts from *ob/ob* diabetic and from cardiac-specific Serca2a^−/−^ mice. Similarly, NFATc increased resistin expression in myocytes cultured in low glucose while the NFATc inhibitor VIVIT blocked glucose-induced resistin expression, suggesting that hyperglycemia/diabetes induces resistin expression possibly through NFATc activation. Interestingly, overexpression of Serca2a or VIVIT mitigated glucose-stimulated resistin and NFATc expression and enhanced AMPK activity, a downstream target of resistin signaling. NFATc direct activation of resistin was verified by resistin promoter luciferase activity and chromatin-immunoprecipitation analysis. Interestingly, activation of Serca2a by a novel agonist, CDN1163, mirrored the effects of AAV9-Serca2a gene transfer on resistin expression and its promoter activity and AMPK signaling in diabetic mice. These findings parse a role for Ca^2+^ in resistin transactivation and provide support that manipulation of Serca2a-NFATc-Resistin axis might be useful in hyper-resistinemic conditions.

## Introduction

Obesity and diabetes are widely recognized as major risk factors for cardiac dysfunction and heart failure (HF)^1,2^. Cardiovascular disease, including HF, is the major cause of morbidity and mortality in type 2 diabetes patients^3–5^. Experimental studies in diabetic animal models and extensive clinical trials have supported the concept of diabetic cardiomyopathy - a clinical myocardial condition distinguished by ventricular dysfunction that occurs independently of coronary artery disease and hypertension^6–9^. Although the pathogenesis of diabetic cardiomyopathy is unclear, potential mechanisms include insulin resistance, altered substrate metabolism, mitochondrial dysfunction, increased oxidative stress and disturbances in adipokines secretion and signaling^7,9,10^. These abnormalities lead to impaired calcium homeostasis resulting in lusitropic and inotropic defects.

Resistin, a cysteine-rich hormone secreted primarily by rodent fat cells, was postulated to be implicated in obesity, insulin resistance and diabetes^11,12^. Recombinant resistin protein was found to impair insulin action in normal mice and cultured adipocytes and immuno-neutralization of resistin improved insulin action in mice with diet-induced obesity^12^. Overexpression of resistin in metabolically healthy mice led to insulin resistance and dysregulated lipid metabolism with increased accumulation of triglycerides and cholesterol^13,14^. Plasma resistin levels were increased in *db/db, ob/ob* and diet-induced obese mice^12^, while resistin mRNA levels in adipose tissue of obese rodents were often found to be decreased^15^. Notably, mice lacking resistin have improved glucose tolerance compared with wildtypes both in diet-induced obesity^16^ and in *ob/ob* mice^17^, suggesting a role for resistin in insulin resistance and hyperglycemia associated with obesity. However, the pathophysiological role of resistin in humans has been questioned because the human homologue of resistin is only 59% identical to mouse resistin at the amino acid level and the source of resistin appears to differ between humans and mice^12,18^. Unlike mice, resistin in humans is undetectable in adipocytes but highly expressed in macrophages. However, emerging evidence suggests that cardiovascular disease is accompanied by changes in resistin levels. For example, plasma resistin levels were elevated in female patients with coronary heart disease^19^ and were independently correlated with new onset heart failure^20^. Elevated levels of resistin were observed in the serum of obese and type-2 diabetic patients and were considered to be a predictor of poor prognosis in patients with cardiovascular disease^21–24^. Studies have also reported higher resistin levels in patients with acute myocardial injury in conjunction with diabetes as compared to non-diabetics^25^, while higher serum levels of resistin were elevated in patients with HF^24^ and demonstrated to be a predictor of the presence and severity of coronary artery disease^26^ and positively related to the severity and incidents of HF hospitalization^20,22^. Collectively, these findings strongly suggest a pivotal role for resistin in heart disease.

We have recently reported that cardiac tissue from human HF patients and from type 1 and 2 diabetic experimental animals expressed high levels of resistin^27^, and adenoviral overexpression of resistin induced hypertrophy, contractile dysfunction with impaired Ca^2+^ handling^27^, and insulin resistance in isolated cardiomyocytes^28^. We have subsequently shown that long-term cardiac-specific overexpression of resistin *in vivo* using adeno-associated virus serotype 9 significantly decreased left ventricular contractility and induced a complex phenotype of oxidative stress, fibrosis, apoptosis and myocardial remodeling in normal rats^29^, very much resembling a diabetic cardiomyopathy phenotype.

This study was designed to address two questions: (1) what drives resistin up-regulation in diabetic hearts; and (2) does restoration of Ca^2+^ homeostasis attenuates its transactivation. Here we provide evidence that normalization of diabetes-induced Ca^2+^ dyshomeostasis in diabetic hearts, through restoration of the sarco/endoplasmic reticulum Ca^2+^-ATPase (Serca2a) function, significantly suppressed resistin expression via inhibition of NFATc and enhanced the activity of AMP-activated protein kinase (AMPK), a downstream target of resistin signaling. Pharmacologically activating Serca2a with CDN1163 – our newly discovered Serca2 allosteric modulator^30^, mirrored the effects of Serca2a gene transfer on NFATc expression, resistin expression and its promoter activity, and AMPK signaling *in vitro* and *in vivo* in *ob/ob* diabetic mice. These findings parse a role for Ca^2+^ in resistin transcriptional activation and provide support that pharmacological manipulation of Serca2a-NFATc-Resistin axis may have great potential impact for translation.

## Results

### Upregulation of resistin in diabetic hearts

**Figure 1:**
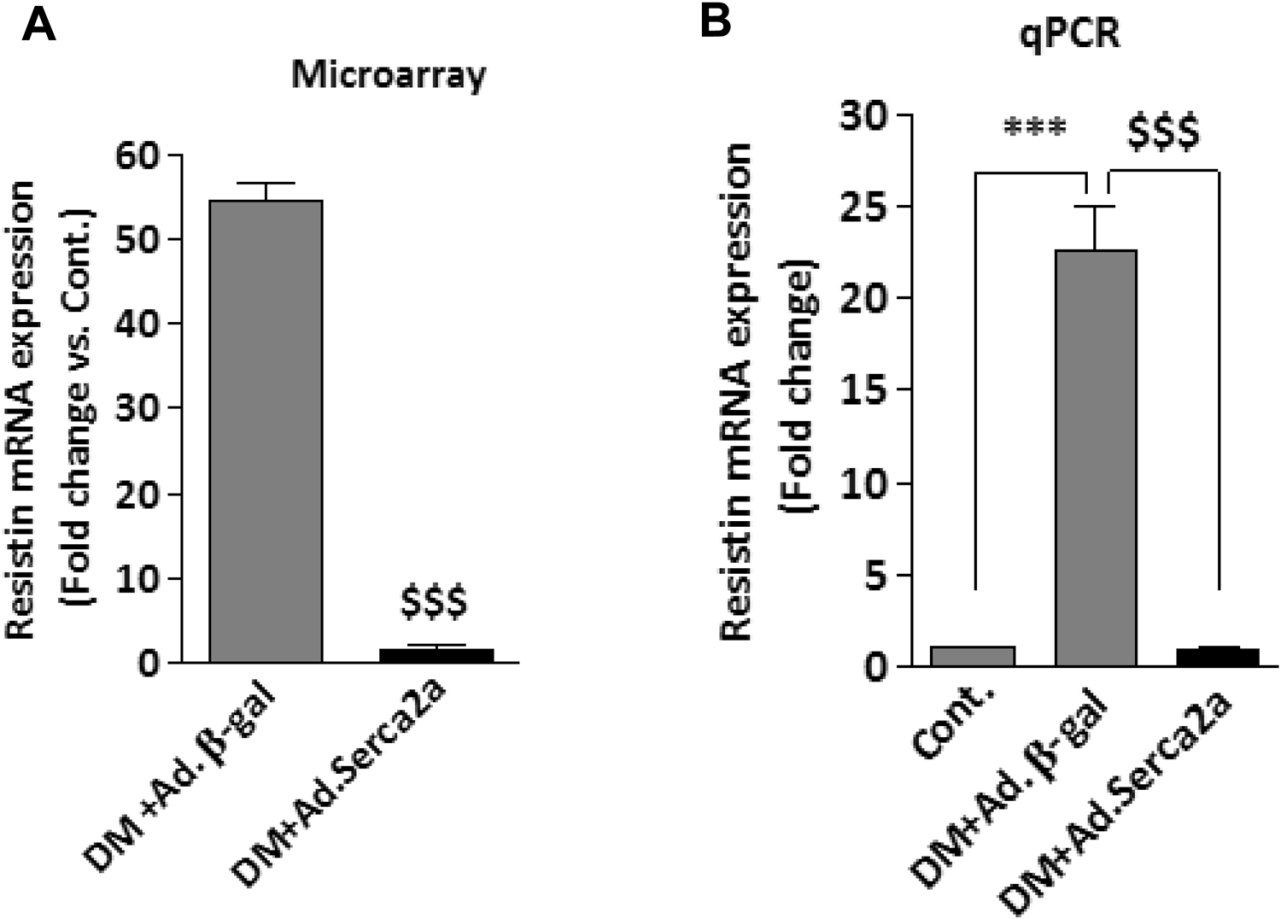
Upregulation of resistin in diabetic hearts. Microarray (**A**) and q-PCR (**B**) analysis of resistin mRNA expression in diabetic mouse hearts without and with adenovirus mediated Serca2a overexpression. Microarray analyses are detailed in ref.^31^. ^$$$^*p* < *0.001* vs Ad.βgal; ***p < *0.001* vs Cont.

Diabetes is associated with impaired cardiac function in both humans and animals. Diabetic cardiomyopathic hearts are generally characterized by diastolic dysfunction associated with abnormal calcium (Ca^2+^) handling and a decrease in Sarco/endoplasmic reticulum Ca^2+^-ATPase (Serca2a) expression^31,32^. We have previously reported that diabetic animal and human failing hearts expressed high levels of resistin^27^. We have observed that restoration of Serca2a function in diabetic mice hearts using *in vivo* adenoviral gene transfer of Serca2a significantly reduced resistin expression to control levels (Fig. 1). Since Serca2a plays an important role in maintaining Ca^2+^ homeostasis in cardiac myocytes, this finding suggests that intracellular Ca^2+^ may potentially mediate Serca2a-produced resistin down-regulation. How diabetes induces and Serca2a reduces resistin expression in cardiomyocytes and whether this is regulated at the transcriptional level is currently unknown.

### Glucose upregulates resistin expression through activation of NFATc *in vitro*

**Figure 2:**
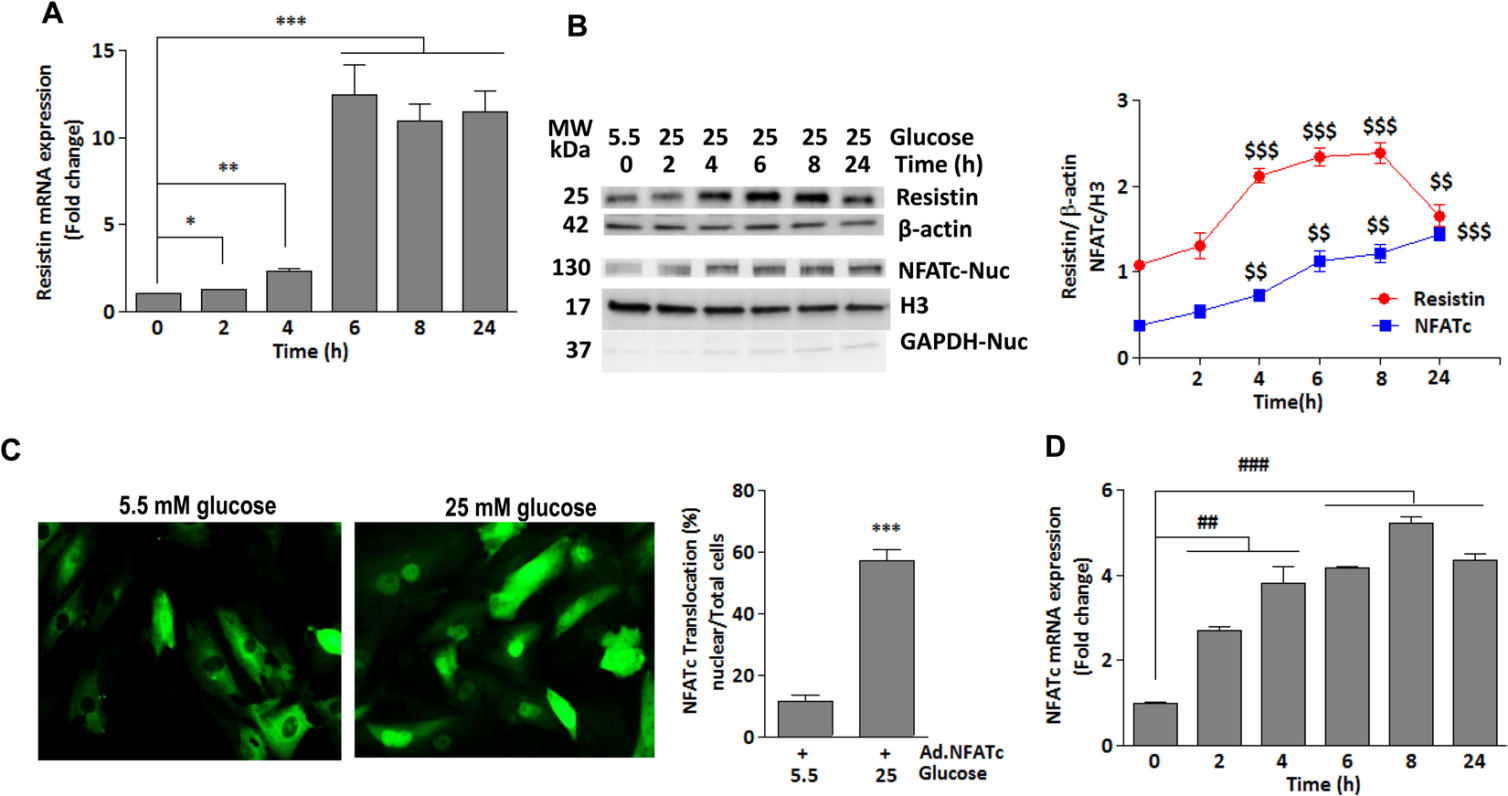
Glucose upregulates resistin expression through activation of NFATc *in vitro*- H9c2 cells were treated with high glucose concentration (25 mM vs. 5.5 mM) for the indicated times. The mRNA expression of resistin (**A**) and NFATc (**D**) was analyzed by q-PCR. 18S rRNA was used as an internal control (**A**,**D**). (**C**) Representative fluorescence microscopic images of nuclear translocation of NFATc-GFP overexpressed in H9c2 cells for 48 hours and then stimulated with high glucose for an additional 4 hours. Quantification of % nuclear import in cells from 5–6 different images is shown (**C**) ***p < *0.001* vs low glucose. Western blotting analysis and densitometry quantification of resistin and nuclear NFATc protein expressions (**B**) are shown. β-actin and H3 were used as internal controls, respectively (**B**). GAPDH also verified purity of nuclear fraction (**B**). The data are mean ± SEM of at least three experiments in triplicates. *p < *0.05* 0 hr vs 2 hrs; **p < *0.01* 0 hr vs 4 hrs ***p < *0.001* 0 hr vs 6–24 hrs; ^$$^*p* < *0.01 and* ^$$$^*p* < *0.001* vs 0 hr; ^##^*p* < *0.001* 0 hr vs 2–4 hrs, ^###^*p* < *0.001* 0 hr vs 6–24 hrs.

To investigate the mechanism underlying the role of diabetic condition in modulating resistin expression, mRNA and protein expressions were measured at different time points in H9c2 cells stimulated with high glucose concentration (25 mM vs. 5.5 mM as a control). A significant increase in resistin expression at both mRNA (Fig. 2A) and protein (Fig. 2B) levels were observed in myocytes treated with high glucose as compared to low glucose. Resistin mRNA expression gradually increased in response to high glucose concentration (25 mM) reaching a maximum level after 6 hrs (Fig. 2A). Resistin protein levels began to increase 4 hrs after stimulation and reached maximal expression after 8hrs (Fig. 2B). These data are in agreement with our earlier findings that hyperglycemic diabetic mice showed increased resistin heart expression (Fig. 1)^27^. Using *In Silico* analysis, we identified NFATc as potential transcription factor that regulates resistin transcription. This is in agreement with a previous report that showed NFATc also regulates resistin in adipocytes^33^. To examine whether hyperglycemia affects NFATc activity, H9c2 cells, infected with an adenovirus encoding NFATc (Ad.NFATc) for 24 hours then treated with high glucose, showed significant NFATc nuclear translocation, indicative of its activation (Fig. 2C). The nuclear expression of NFATc mRNA (Fig. 2D) and protein (Fig. 2B) gradually increased until it reached maximal levels after 8 hrs. Interestingly, NFATc expression pattern highly resembled that of resistin expression, suggesting that hyperglycemia-induced resistin upregulation may be a result of aberrant activation of NFATc, which may transcriptionally regulate resistin expression.

### Glucose-induced resistin expression and NFATc activation are calcium-dependent

**Figure 3:**
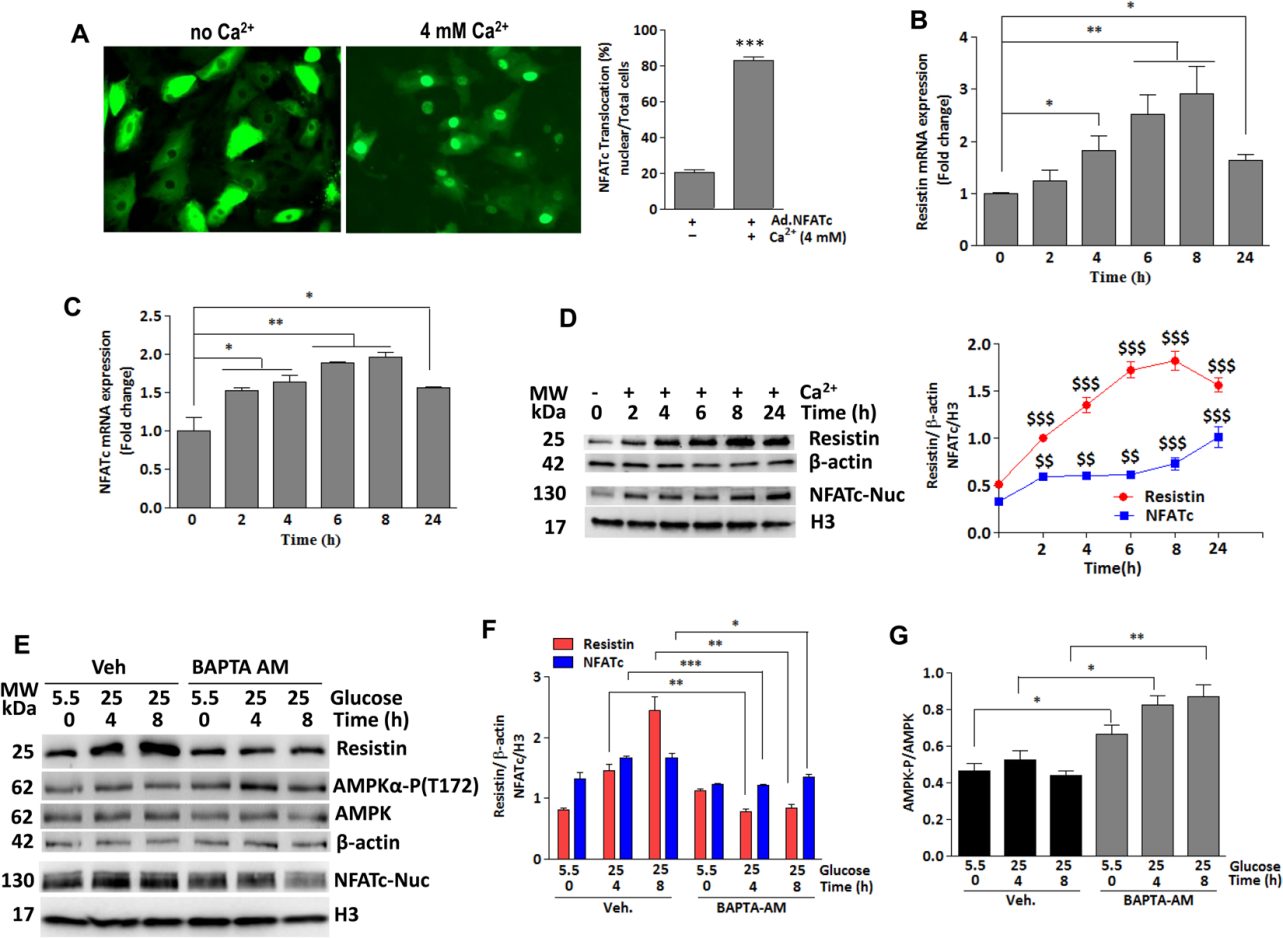
Calcium drives glucose-induced expression of resistin and NFATc. (**A**) Representative fluorescence microscopic images of nuclear translocation of NFATc-GFP overexpressed in H9c2 cells for 48 hours and then stimulated with Ca^2+^ for an additional 4 hours. Quantification of % nuclear import in cells from 5–6 different images is shown (**A**). ***p < *0.001* vs no Ca^2+^. H9c2 cells were treated with 4 mM Ca^2+^ for the indicated times. The mRNA expression of (**B**) resistin (cytosolic fraction) and (**C**) NFATc (nuclear fraction) was analyzed by q-PCR. 18S rRNA was used as an internal control. The data are mean ± SEM of three different experiments in triplicates. *p < *0.05* 0 hr vs 4 and 24 hrs; **p < *0.01* 0 hr vs 6–8 hrs (**B**); *p < *0.05* 0 hr vs 2–4 hrs, and 24 hrs; **p < *0.01* 0 hr vs 6–8 hrs (**C**). Western blotting analysis and densitometry quantification of resistin and nuclear NFATc proteins expression are shown (**D**). ^$$^*p* < *0.01* and ^$$$^*p* < *0.001* vs 0 hr. To verify Ca^2+^ specificity, H9c2 cells were treated with BAPTA-AM (2 μM) and stimulated with high glucose concentration (25 mM vs. 5.5 mM) for the indicated times. The protein expression of resistin, nuclear NFATc and Phosphorylation of AMPKα were analyzed by western blotting (**E**) with densitometry quantification shown in (**F** and **G**, respectively). Phosphorylation of AMPKα is presented as phospho-AMPK/AMPK ratio. H3 was used as an internal control for NFATc nuclear expression, β-actin was used as an internal control for the other proteins; (**E**) *p < 0.05, **p < *0.01* and ***p < *0.001* Veh vs BAPTA-AM at the indicated hrs. (**G**) *p < *0.05* and **p < *0.01* Veh vs BAPTA-AM at the indicated hrs.

A dysfunctional Serca2a leads to altered intracellular Ca^2+^ handling in diabetic cardiac myocytes^31^. Our earlier observation that Serca2a overexpression in diabetic hearts led to down-regulation of resistin (Fig. 1) strongly suggests that intracellular Ca^2+^ might be involved in its transcriptional pathway. To this end, H9c2 cells infected with Ad.NFATc for 24 hours then stimulated with Ca^2+^ (4 mM)^34^ for 4 hours, showed significant NFATc nuclear translocation (Fig. 3A). Resistin and nuclear NFATc expressions were then analyzed in H9c2 cells treated with Ca^2+^ (4 mM) for the indicated time (Fig. 3). Both resistin and nuclear NFATc showed significant parallel increases in both mRNA (Fig. 3B,C) and protein (Fig. 3D) levels compared to non-treated. The expression of both molecules appears to be time-dependent as it gradually increased in response to Ca^2+^ until it reached maximum levels after 8hrs (Fig. 3). These findings clearly support a role for Ca^2+^ in the activation of resistin-NFATc and strongly suggest that impaired Ca^2+^ homeostasis, generally found in diabetic heart, is potentially responsible for the observed upregulation of these molecules. The concomitant Ca^2+^-stimulated expression increases in resistin and nuclear NFATc further suggest a transcriptional link between them.

To further verify the specificity of Ca^2+^ effect, we used the cell-permeant selective Ca^2+^ chelator BAPTA-AM to control the level of intracellular Ca^2+^. In this regard, high glucose (25 mM vs. 5.5 mM) treatment failed to alter resistin or nuclear NFATc expression in BAPTA-AM treated H9c2 cells (Fig. 3E,F). We have previously demonstrated that resistin promotes cardiac hypertrophy and insulin resistance through inhibition of AMP-activated protein kinase (AMPK) activity^28^. To further demonstrate the functional significance of Ca^2+^ inhibition-conferred protection against high glucose stimulation we analyzed the activity of AMPK, a downstream target of resistin. Accordingly, resistin and NFATc reduction by BAPTA-AM led to a significant elevation in AMPKα phosphorylation (Fig. 3E,G). These findings strongly suggest that glucose-induced resistin expression and NFATc translocation are Ca^2+^-mediated.

### Inhibition of NFATc attenuates glucose and Ca^2+^-induced expression of resistin

**Figure 4:**
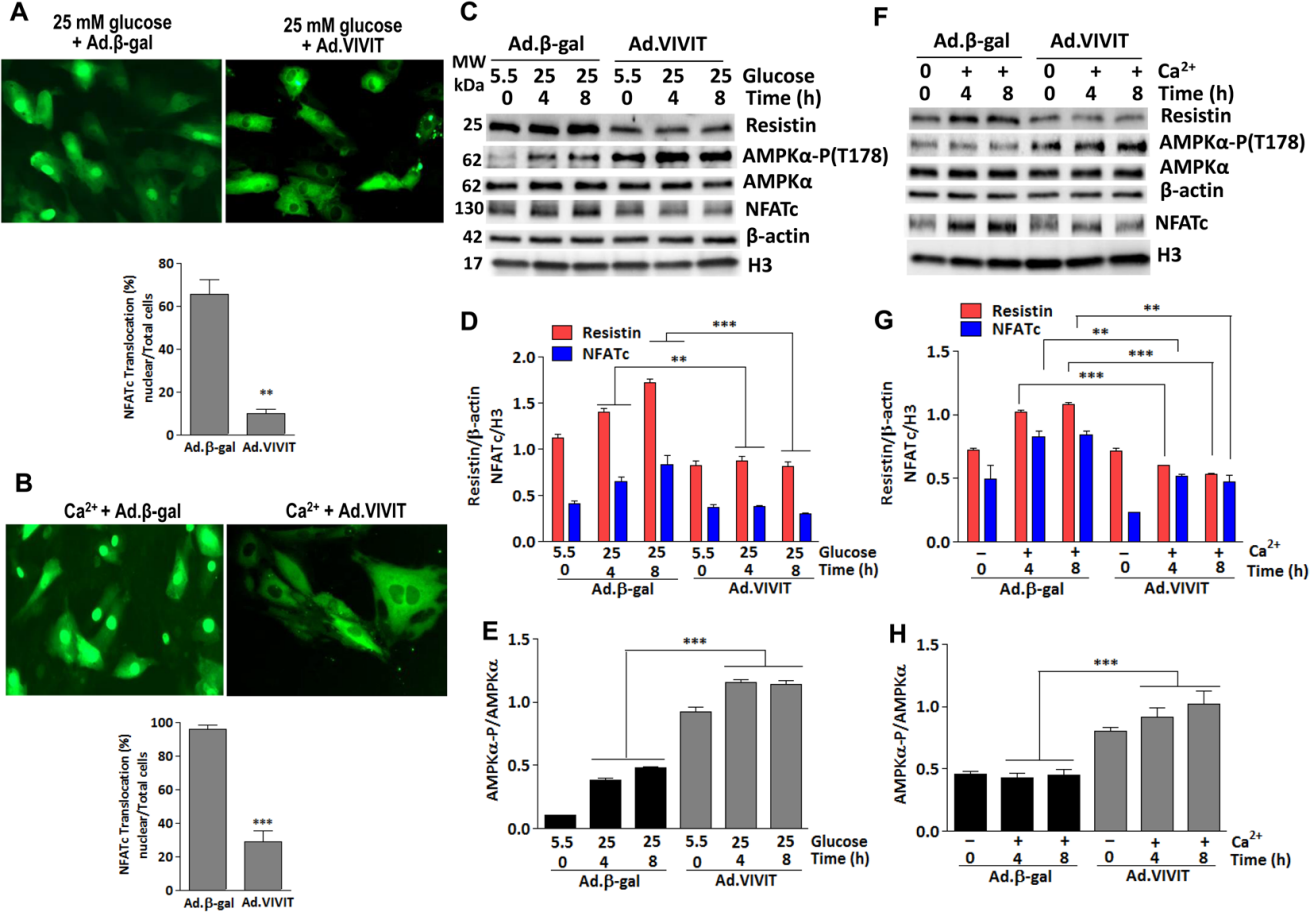
Glucose and Ca^2+^ fail to induce resistin expression in NFATc-inhibited cells-Representative fluorescence microscopic images of H9c2 cells co-infected with Ad.NFATc-GFP and Ad.VIVIT for 48 hours then treated with either high glucose (**A**) or Ca^2+^ (**B**) for 4 hours and NFATc-GFP nuclear translocation was visualized and quantified in more than 5 images in each condition (**A** and **B**, respectively). **p < *0.01* and ***p < *0.001* vs Ad.βGal. (**C**–**H**) VIVIT-expressing H9c2 cells were stimulated with 25 mM glucose (**C**) or 4 mM Ca^2+^ (**F**) for the indicated times. The protein expression of resistin and NFATc (nuclear), and phosphorylation of AMPKα were analyzed by western blotting (**C**,**F**) and densitometry quantifications were determined (**D**,**G**, and **E**,**H**, respectively). The phosphorylation of AMPK status is reported as phospho-AMPK/AMPK ratio. H3 was used as an internal control for NFATc nuclear expression, β-actin was used as an internal control for the other proteins. The data are mean ± SEM of three experiments in triplicates. **p < *0.01* and ***p < *0.001* Ad.βGal vs Ad.VIVIT.

The above findings provide evidence that high glucose and Ca^2+^stimulate resistin expression potentially through increased NFATc activation. To validate that NFATc mediates glucose and Ca^2+^ effects on resistin upregulation, we infected H9c2 cells for 24 hrs without or with an adenovirus encoding VIVIT (a peptide inhibitor of NFATc) to inhibit NFATc then treated the cells with either high glucose (25 mM vs. 5.5 mM) or Ca^2+^ (4 mM) as indicated (Fig. 4). As expected, both glucose and Ca^2+^ stimulation failed to induce NFATc nuclear translocation (Fig. 4A,B, respectively). Similarly, in the presence of VIVIT, glucose and Ca^2+^ failed to induce resistin and nuclear NFATc protein expression (Fig. 4C,D) and (Fig. 4F,G), respectively. Furthermore, addition of VIVIT reversed and increased resistin-induced dephosphorylation of AMPKα (Fig. 4C,E,F,H). These data demonstrate that NFATc mediates the upregulation of resistin expression induced by high glucose and Ca^2+^.

### Serca2a overexpression downregulates NFATc and suppresses resistin expression *in vitro*

**Figure 5:**
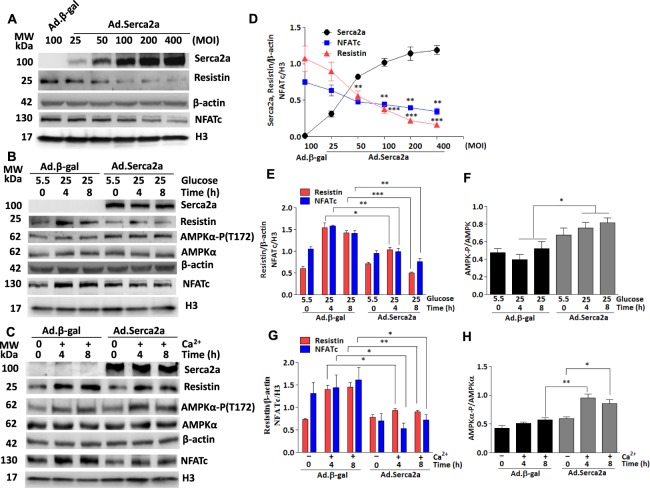
Serca2a overexpression suppresses high glucose induced-resistin and NFATc expressions and enhanced AMPK activation. H9c2 cells were infected with increasing multiplication of infection (MOI) of Ad.Serca2a. The expression of Serca2a, resistin and NFATc (nuclear) was analyzed by western blotting (**A**) and densitometry quantifications were determined (**D**). **p < *0.01* and ***p < *0.001* vs baseline Ad.βgal. Serca2a-overexpressing H9c2 cells (MOI:50) were treated with high glucose concentration (25 mM vs. 5.5 mM) for the indicated times. The expression of resistin, nuclear NFATc and phosphorylation of AMPKα were analyzed by western blotting (**B**) and densitometry quantifications were obtained (**E**,**F**). Serca2a-overexpressing H9c2 cells were treated with 4 mM Ca^2+^ for the indicated times and the expression of resistin, nuclear NFATc, and phosphorylation of AMPKα were analyzed by western blotting (**C**) and densitometry quantification (**G**,**H**). The phosphorylation of AMPK status is presented as phospho-AMPK/AMPK ratio. β-actin was used as an internal control. The data are mean ± SEM of at least three experiments in triplicates. *p < *0.01*, **p < *0.01* and ***p < *0.001* Ad.Serca2a vs Ad.β-gal at the indicated times.

Since Serca2a plays a critical role in controlling the cytosolic (i.e. diastolic) concentration of Ca^2+^ in cardiac myocytes, we therefore asked the question as to whether Serca2a activity would also regulate resistin transcriptional activity. To address this issue, we infected H9c2 cells with increasing doses of Serca2a (i.e. Ad.Serca2a; MOI in Fig. 5) and the expression of Serca2a, resistin and nuclear NFATc were analyzed by immunoblotting. Serca2a overexpression dose-dependently reduced NFATc protein nuclear accumulation and resistin protein expression (Fig. 5A), further supporting a role for Ca^2+^ in the upregulation of NFATc and resistin and suggests that restoration of diabetes-induced Serca2a dysfunction represses NFATc-driven resistin transcription.

To further characterize the effect of Serca2a on NFATc-resistin under diabetic conditions, we explored the effects of glucose on myocytes overexpressing Serca2a. H9c2 cells were infected with Ad.βgal (control) or Ad.Serca2a and stimulated with high glucose (25 mM vs. 5.5 mM) for the indicated time. Again, Serca2a overexpression significantly attenuated glucose-promoted upregulation of resistin and nuclear NFATc expression (Fig. 5B,E). The role of Serca2a in resistin-mediated signaling was functionally tested by determining the activation of AMPKα. Interestingly, Serca2a expression significantly increased AMPKα phosphorylation in glucose-stimulated cells (Fig. 5B,F), suggesting that Serca2a restoration in a diabetic environment is able to reverse the effects of resistin on AMPKα activity.

To further explore the effects of Serca2a on resistin expression and its associated molecular pathways in conditions of Ca^2+^ overload, as generally observed in diabetic hearts, H9c2 cells were infected with Ad.βgal (control) or Ad.Serca2a then treated with Ca^2+^ (4 mM)^34^ for the indicated times (Fig. 5C). Interestingly, Ca^2+^-induced resistin and nuclear NFATc expressions were significantly reduced in Serca2a-overexpressing cells as compared to control Ad.βgal-infected (Fig. 5C,G). Likewise, Serca2a expression significantly promoted AMPKα phosphorylation in Ca^2+^-treated cells (Fig. 5C,H), inversely dovetailing the expression of resistin. In aggregate, these data demonstrate that improved Ca^2+^ homeostasis following Serca2a expression in myocytes reduced the diabetic effects of high glucose and Ca^2+^ on resistin and NFATc activity as well as normalized the activity of AMPKα.

### *In-vivo* cardiac-specific Serca2a gene transfer in diabetic hearts downregulates resistin expression and NFATc activation

**Figure 6:**
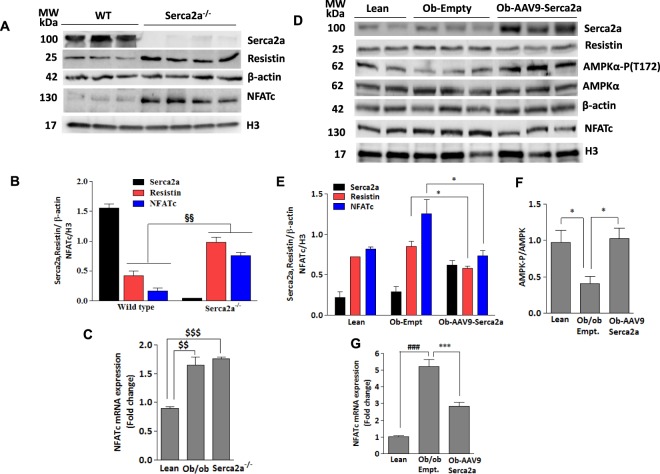
Serca2a knockdown increases resistin and NFATc expression in adult mice. Heart tissues of Serca2a knockout or wildtype mice were analyzed for Serca2a, resistin and nuclear NFATc expressions by western blotting (**A**) with densitometry quantification shown (**B**). ^**§§**^*p* < *0.01* vs wildtype. The expression of nuclear NFATc mRNA was analyzed in hearts from diabetic *ob/ob* and Serca2a-deficient mice (Serca2a^−/−^) (**C**). *Ob/ob* diabetic mice were infected with AAV9-Serca2a or AAV9-empty vectors and protein expression of resistin, nuclear NFATc, and phosphorylation of AMPKα were analyzed by western blotting (**D**) with densitometry quantifications shown (**E**,**F**). The expression of nuclear NFATc mRNA was analyzed in AAV9-Serca2a or AAV9-empty vector infected *ob/ob* mice hearts (**G**). 18S rRNA was used as an internal control for q-PCR. The phosphorylation of AMPK status is presented as phospho-AMPK/AMPK ratio. H3 and β-actin was used as corresponding internal controls for western blotting. The data are mean ± SEM of three experiments from 5–6 animals. ^$$^*p* < *0.01 lean vs Ob/ob;* ^$$$^*p* < *0.001* lean vs Serca2a^−/−^ (**C**) *p < *0.05* lean vs ob-Empt, and ob-Empt vs AAV9-Serca2a (E and F); ***p < *0.001* *ob/ob*-Empt vs AAV9-Serca2a (**G**) ^###^*p* < *0.001* lean vs *ob/ob*-Empt (**G**).

Diabetes progressively leads to decreased levels of Serca2a and impaired Ca^2+^ homeostasis in cardiomyocytes. *Ob/ob* mice have been shown to exhibit reduced Serca2a expression^32^. To further confirm our *in vitro* findings and the role of Serca2a on resistin and NFATc expression *in vivo*, we first determined the protein expression of resistin and nuclear NFATc in cardiac-specific Serca2a knockout mice hearts by western blotting. Ablation of Serca2a significantly increased the expression of resistin and nuclear NFATc in these mice compared to wildtype (Fig. 6A,B). Similar to protein levels, NFATc mRNA also increased in Serca2a-deficient as well as diabetic hearts (Fig. 6C).

To further confirm the role of Serca2a in the regulation of NFATc and resistin expression *in vivo*, we tail-vein injected *ob/ob* mice with adeno-associated serotype 9 (AAV9.Serca2a) or empty vector (AAV9.Empt) for 12 weeks. AAV9.Serca2a overexpression significantly reduced the expression of resistin and NFATc nuclear accumulation (Fig. 6D,E) and reconciled diabetes-associated decrease in AMPKα phosphorylation (Fig. 6D,F). Similar to its effects on NFATc protein expression, Serca2a overexpression in *ob/ob* hearts reduced diabetes-induced NFATc mRNA levels (Fig. 6G). Taken together, these results demonstrate a critical role for Serca2a in suppressing the activity of resistin and reversing its downstream signaling pathways, further supporting the beneficial role of Serca2a restoration in diabetic hearts.

### NFATc induces resistin expression and activates resistin promoter

**Figure 7:**
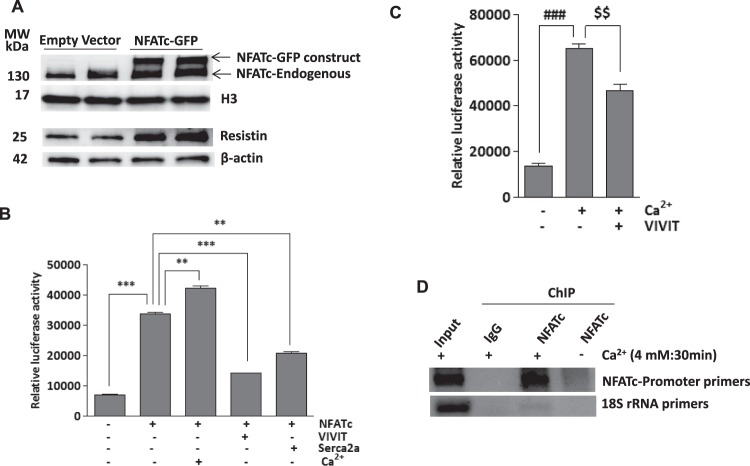
NFATc induces resistin expression and activates resistin promoter activity- H9c2 cells were transiently transfected with Ad.NFATc-GFP or Ad.empty vectors for 48hrs and nuclear NFATc and resistin protein expression was measured by western blotting with H3 or β-actin used as loading controls, respectively (**A**). Myocytes were transduced with Ad.NFATc and resistin promoter-mediated luciferase activity was measured in the presence of VIVIT, Serca2a or Ca^2+^ (4 mM) (**B**). Myocytes transduced with Ad.VIVIT were stimulated with 4 mM Ca^2+^ and resistin promoter-mediated luciferase activity was measured as indicated (**C**). Chromatin immunoprecipitation (ChIP) assay was performed to determine binding of NFATc to resistin transcription loci in the NFATc overexpressing or control H9c2 cells stimulated with Ca^2+^ (4 mM) for 30 min. The agarose gel picture of the PCR products shows relative binding of NFATc to a specific region of resistin promoter, precipitated with either NFATc or IgG antibody. 18S rRNA was used as control (**D**). **p < *0.01* NFATc vs Ca^2+^ and Serca2a; ***p < *0.001* Cont vs NFATc, NFATc vs VIVIT (**B**). ^$$^*p* < *0.001* Ca^2+^ vs VIVIT ^###^*p* < *0.01* vs Ca^2+^.

Given the above findings that NFATc increased resistin expression in cardiac myocytes, we sought to determine whether NFATc regulates resistin expression at the transcriptional level. Transient overexpression of NFATc (NFATc-GFP) in H9c2 cells led to a significant induction in the expression of resistin protein (Fig. 7A). We then tested if overexpression of NFATc in cardiomyocytes could stimulate and drive resistin promoter-dependent luciferase activity. Ca^2+^ treatment as well as NFATc overexpression markedly increased resistin-luciferase activity, which was prevented by VIVIT or Serca2a overexpression (Fig. 7B). Interestingly, the effects of Ca^2+^ appears to be additive to those evoked by NFATc, suggesting that other NFATc-independent mechanisms might be involved in resistin gene promoter activity. This is further confirmed when resistin-luciferase activity was determined in myocytes treated with Ca^2+^ in the absence of ectopic NFATc expression (Fig. 7C). Again, Ca^2+^-induced luciferase activity is significantly decreased by VIVIT expression although not to control levels (Fig. 7C), further suggesting that Ca^2+^-driven resistin promoter activity may be mediated by additional transcriptional factors other than NFATc.

The DNA sequence analysis of the resistin promoter revealed putative binding sites for NFATc. The role of NFATc in resistin trans-activation was further examined in H9c2 cells treated with Ca^2+^ and subjected to chromatin immunoprecipitation (ChIP) using a specific antibody against NFATc. Binding of NFATc to the resistin promoter was verified by PCR using resistin promoter-specific primers. The ChIP analysis showed enhanced binding of NFATc to the resistin promoter upon stimulation with Ca^2+^ compared to control. Isotype-matching IgG and amplification of the 18 S rRNA were used as controls (Fig. 7D). These findings clearly establish a role for NFATc as a transcriptional regulator of resistin driven by changes in intracellular Ca^2+^ levels.

### Pharmacological activation of Serca2a with novel small molecule allosteric modulator inactivates NFATc and downregulates resistin expression

**Figure 8:**
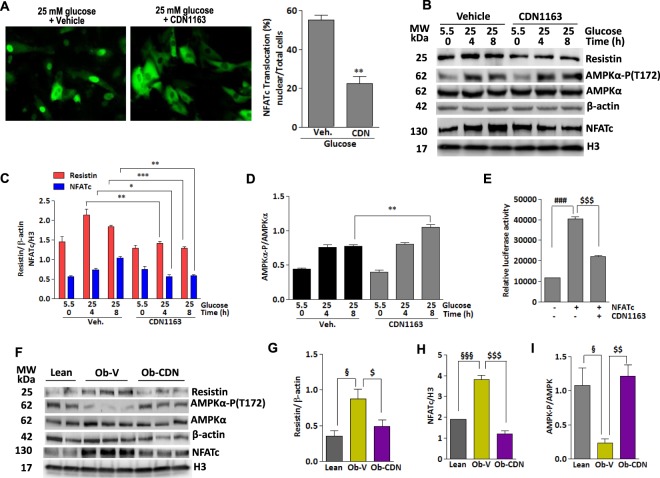
Pharmacological activation of Serca2a with small molecule CDN1163 inactivates NFATc and reduces resistin expression. (**A**) Representative fluorescence microscopic images of H9c2 cells transfected with NFATc-GFP for 24 hours and then incubated with CDN1163 (10 μM) or vehicle for an additional 24 hours. Cells were then stimulated with high glucose for 4 hours and nuclear import of NFATc-GFP was visualized and quantified in more than 5 different images per condition (**A**). **p < *0.01* CDN1163 vs Veh. (**B**) H9c2 cells were treated with CDN1163 and stimulated with high glucose concentration (25 mM vs. 5.5 mM) for the indicated times. The expression of resistin, nuclear NFATc proteins, and phosphorylation of AMPKα were analyzed by western blotting (**B**) with densitometry quantifications determined (**C**,**D**). Resistin promoter-mediated luciferase activity was measured in H9c2 cells overexpressing NFATc then treated with CDN1163 for 24 hours (**E**). *Ob/ob* mice were injected with CDN1163 (Ob-CDN) or vehicle (Ob-V) for 4 weeks and heart tissues were analyzed for the expression of resistin, nuclear NFATc and phosphorylation of AMPKα by western blotting (**F**) with densitometry quantifications shown (**G**–**I**, respectively) with H3 or β-actin used as corresponding internal controls. The phosphorylation of AMPK status is presented as phospho-AMPK/AMPK ratio. The data are mean ± SEM of three experiments from 4–5 animals. *p < *0.05*, **p < *0.01* and ***p < *0.001* veh vs CDN1163 at the indicated times (**C**,**D**); ^###^*p* < *0.001* Cont vs NFATc; ^$$$^*p* < *0.001* NFATc vs CDN1163; ^§^*p* < *0.05* and ^§§§^*p* < *0.001* lean vs Ob-V; ^$^*p* < *0.01*, ^$$^*p* < *0.01* and ^$$$^*p* < *0.001* Ob-V vs CDN1163.

The studies above established a role for Serca2a gene expression in the control of resistin transcription. We next wanted to explore if pharmacological activation of Serca2a would evoke similar dynamics to Serca2a gene transfer as outlined above. To this end, we took advantage of our newly discovered small molecular Serca2 allosteric modulator, CDN1163. We have recently demonstrated that CDN1163 directly binds to and activates Serca2 Ca^2+^-ATPase activity^30^, leading to increased endoplasmic reticulum Ca^2+^ load and major improvement in Ca^2+^ homeostasis in *ob*/*ob* diabetic mice)^30^. Here, we demonstrate that CDN1163 markedly abolished glucose-stimulated NFATc nuclear translocation (Fig. 8A). Likewise, in the presence of CDN1163 high glucose-induced resistin and nuclear NFATc expression were significantly reduced (Fig. 8B,C) while the phosphorylation of AMPKα is increased in a time-dependent manner (Fig. 8B,D), indicating that CDN1163-mediated activation of Serca2a function affects resistin and NFATc expression patterns in a similar manner as Serca2a gene transfer. Interestingly, CDN1163 also reduced NFATc-mediated resistin promoter luciferase activity (Fig. 8E).

We next wanted to test if CDN1163 would also evoke similar effects on resistin *in vivo*. *Ob/ob* mice were treated with CDN1163 for 4 weeks as indicated in the methods and the cardiac levels of NFATc and resistin were then analyzed by immunoblotting. Indeed, hearts from CDN1163-treated mice showed significant decrease in resistin (Fig. 8F,G) and nuclear NFATc (Fig. 8F,H) protein expression compared with vehicle-treated mice. Consistent with the decline in resistin expression, CDN1163 treatment increased AMPKα activity/phosphorylation in *ob/ob* mice hearts compared to vehicle-treated (Fig. 8F,I). Altogether, these results demonstrate that CDN1163 is able to regulate resistin expression *in vitro* and *in vivo*, validating the pharmacological activation of Serca2a as a treatment for hyper-resistinemic conditions.

## Discussion

Resistin, a cysteine-rich hormone secreted by rodent fat cells, was found to impair glucose metabolism and insulin action in mouse models of obesity and cultured adipocytes. A variety of cardiovascular effects of resistin were reported since its discovery in 2001, such as the induction of endothelial dysfunction and the promotion of ischemia-reperfusion myocardial injury^35,36^. Its role in cardiac function in the diabetic heart remains obscure; however, emerging lines of evidence strongly indicate that hyper-resistinemia may contribute to the impairment of cardiac contractility and development of diabetic cardiac dysfunction. We have demonstrated that cardiac tissues from type 1 diabetic mice and type 2 diabetic humans and rats express elevated levels of resistin^27^. We have subsequently shown that resistin overexpression induced cardiac hypertrophy with impaired Ca^2+^ handling, and insulin resistance in isolated cardiomyocytes^28^ while long-term cardiac-specific overexpression of resistin *in vivo* significantly decreased left ventricular contractility and induced a complex phenotype of oxidative stress, fibrosis, apoptosis and myocardial remodeling in normal rats, producing a phenotype resembling diabetic cardiomyopathy^29,37^. Interestingly, myocardial-targeted restoration of Serca2a function in diabetic hearts markedly normalized resistin expression in these hearts to control levels (Fig. 1), raising the prospect that measures that underlie resistin repression through Serca2a activation may emerge as a potential objective in the treatment of diabetes-induced heart failure. Our objective in the current studies is to dissect the molecular mechanisms underlying resistin aberrant regulation in diabetic hearts. We provide evidence that diabetes or hyperglycemia induces resistin expression through the activation of the transcription factor NFATc. We also demonstrate that either genetic or pharmacological restoration of Serca2a function represses nuclear NFATc translocation via a Ca^2+^-dependent pathway, resulting in attenuation of resistin expression, and potentially normalization of cardiac function.

Recent studies revealed that several transcription factors are involved in the regulation of resistin expression in adipocytes, however no information is available regarding the molecular drivers of resistin upregulation in diabetic hearts. Treatment with peroxisome proliferator-activated receptor (PPARγ) agonists suppressed resistin expression in 3T3-L1 adipocytes and in white adipose tissue of mice fed with a high fat diet^12^, although a functional PPAR-response element was not found within 6.2 kb upstream of the mouse resistin promoter^38^. However, PPARγ activation was reported to indirectly repress the expression of the resisitn gene via reduction of resistin promoter histone acetylation and recruitment of the adipogenic transcription factor CCAAT/enhancer-binding protein (C/EBPα)^38^. Interestingly, PPARγ has also been reported to induce Serca2b expression in β-cells and prevent their decline in diabetic animals^39^. In 3T3-L1 adipocytes and in THP-1 human monocytes, the expression of resistin was found to be positively associated with the endoplasmic reticulum (ER) stress response. The ER stress transcription factors C/EBPα homologous protein (CHOP) and activating transcription factor-4 (ATF4) are likely involved in its upregulation^40,41^; however, others have observed the opposite effects^42^.

The present studies clearly demonstrate that NFATc is a transcriptional regulator of the resistin gene in the heart. *In silico* analysis identified NFATc as potential transcription factor that regulates resistin promoter, we therefore hypothesized that increased resistin levels seen in diabetic hearts may be a result of aberrant activation of NFATc. Our findings demonstrate that hyperglycemia in cultured myocytes and *in vivo* in cardiac tissues from animal models of diabetes noticeably promoted resistin expression by inducing NFATc activation which was mitigated by the NFATc inhibitor, VIVIT. Furthermore, ectopic expression of NFATc remarkably stimulated resistin expression in myocytes cultured in low glucose. These results were further confirmed by resistin promoter luciferase reporter assays showing NFATc expression trans-activates the mouse resistin gene which was again blocked by VIVIT. *In vivo* ChIP assay further demonstrated that NFATc binds to resistin promoter. This was corroborated by bioinformatics sequence analysis of mouse resistin promoter revealing the presence of at least 2 NFATc binding sites in the 2.5 kb proximal region, strongly suggesting that NFATc may in fact control resistin gene expression by transactivation of resistin promoter. These findings are in agreement with a study reporting the induction of resistin expression in brown adipose tissue of *ob/ob* mice by NFATc signaling^33^. To functionally validate the specificity of NFATc regulation of resistin expression, we examined the activity of AMPK, a downstream target of resistin signaling in cardiomyocytes^28^. Inactivation of NFATc by VIVIT and downregulation of resistin expression led to enhanced phosphorylation of AMPK. Thus, NFATc may contribute to glucose and insulin homeostasis by regulating resistin gene expression and AMPK signaling, given resistin’s involvement in these processes.

Having established a link between NFATc and resistin cardiac transactivation, we then sought to determine if Serca2a expression and normalization of Ca^2+^ homeostasis disrupts resistin gene expression through manipulation of NFATc. Serca2a plays an important role in maintaining Ca^2+^ homeostasis in cardiac myocytes. Impaired Secra2a function causes imbalance in Ca^2+^ cellular compartmentalization that affects a wide variety of cellular and physiological mechanisms, including cell signaling and gene transcription. Thus, a decrease in Serca2a function, as it occurs in failing and diabetic hearts, leads to substantial accumulation of diastolic Ca^2+^ which can activate a Ca^2+^-sensitive signaling system that provides a stimulus for the induction of resistin. We therefore hypothesized that restoration of Serca2a levels and subsequent normalization of Ca^2+^ handling may lead to Ca^2+^-specific disruption of resistin expression. Indeed, Serca2a expression in cultured myocytes and *in vivo* in diabetic hearts reduced nuclear NFATc expression and down-regulated resistin expression, while Serca2a-null mice hearts had increased levels of NFATc. Ectopic Serca2a expression significantly diminished NFATc occupancy at the resistin promoter. Calcineurin, a calcium/calmodulin-dependent phosphatase, and the transcription factor NFATc have long been shown to be implicated in the regulation and control of cardiomyocyte hypertrophy^43^. Activation of calcineurin signaling pathway in cardiac myocytes is believed to initiate hypertrophy via activation of NFATc where it probably interacts with other transcriptional factors and enhancers to modulate gene expression^44–46^. Indeed, cardiomyocytes stimulated with 4 mM Ca^2+^ displayed increased nuclear NFATc and resistin expression while addition of the Ca^2+^ chelator BAPTA-AM attenuated this effect. Furthermore, elevated Ca^2+^ level triggered the binding of NFATc to an NFAT-binding motif within resistin’s promoter and enhanced transcription of resistin, which was abolished by VIVIT. These findings provide clear evidence that Serca2a can target the calcineurin/NFAT pathway through its effect on intracellular Ca^2+^ needed for calcineurin activation. Increased Serca2a activity and/or expression stimulates SR Ca^2+^ uptake thereby diminishing intracellular Ca^2+^ concentration leading to inactivation of the calcineurin/NFAT signaling pathway, and subsequently depression of resistin expression.

We have to point out that since a variety of kinases, transcription factors and signaling cascades are directly activated by Ca^2+^ or use Ca^2+^ as a cofactor^43,46,47^, our studies do not rule out the possible involvement of other Ca^2+^-modulated signaling cascades other than NFATc in the activation of resistin expression. However, the current studies still provide a strong evidence of a Ca^2+^-NFATc axis driving resistin cardiac transactivation.

In the current studies we used 2 complementary approaches to restore Serca2a function, a genetic strategy using AAV9-Serca2a that confers high cardiac tropism, and a pharmacological approach using a newly discovered small molecule activator of Serca2, CDN1163^30^. Interestingly, CDN1163 exhibited similar dynamics to Serca2a gene therapy and markedly attenuated nuclear NFATc and resistin expression, and enhanced AMPK activity *in vitro* and *in vivo* in *ob/ob* diabetic mice. This is of particular interest, as it suggests that developing small molecules that directly target defective endogenous Serca2 enzyme and correct Ca^2+^ imbalance may constitute a novel approach to improve contractility in diabetic hearts. CDN1163’s suppression of NFATc/resistin may as well opens the door for potential anti-hyperresistinemia therapy. We recently demonstrated that CDN1163 attenuated ER stress, ameliorated mitochondrial efficiency, improved glucose and lipid metabolism and normalized ER Ca^2+^ dyshomeostasis *in vivo*, resulting in improved glucose tolerance and metabolic disorders in *ob*/*ob* mice^30^. These observations coupled with our current findings suggest that CDN1163 may confer its broader and protective metabolic benefits through suppression of NFATc-mediated resistin expression and restoration of impaired Serca2a function and activation of AMPK, triggering amelioration of cardiac myocytes metabolism and function. As cardiovascular co-morbidities are common in diabetes, it is possible that pharmacological activities that target Serca2 in the cardiovascular system and in diabetes may have beneficial effects by increasing cardiac contractility and limiting metabolic disorders.

Some limitations to our study need to be acknowledged. First, H9c2 cells were used in the *in vitro* studies. H9c2 is a rat cardiomyoblast cell line derived from embryonic rat heart ventricle and as such they may not faithfully mimic the responses of primary cardiomyocytes. Although their use as stand-alone model may cause certain concerns, we have shown that findings in cardiac tissues *in vivo* strongly recapitulate the cellular findings in H9c2 cells. Furthermore, recent reports have demonstrated that H9c2 cells showed almost identical hypertrophic responses to those observed in primary cardiomyocytes^48^ and are more similar to primary cardiomyocytes with regard to cellular energy metabolism, including ATP levels, and mitochondria bioenergetics, function and morphology^49^, further supporting these cells as a good auxiliary model to cardiomyocytes.

In this study we tested the hypothesis that NFATc differentially regulates resistin activity in cardiac myocytes both *in vitro* and *in vivo*. Despite the fact that four different NFAT isoforms (c1 to c4) are expressed in the heart, with NFATc3 and NFATc4 have established roles in hypertrophic signaling and NFATc1 plays a key role in cardiac development^50–52^, we examined the response of total NFAT and we did not attempt to investigate in depth which isoform specifically activates resistin. However, we found that glucose and Ca^2+^ stimulation is associated with activation and nuclear translocation of NFATc4 but observed no changes in the expression of the other NFAT isoforms, c1, c2 or c3 (not shown). These observations may infer that aberrant activation of NFATc4 is an essential mediator of and likely responsible for resistin upregulation.

We also hypothesized in this study that restoration of Ca^2+^ homeostasis attenuates NFAT-induced resistin transactivation. In cardiomyocytes, intracellular Ca^2+^ concentrations are tightly regulated by a number of Ca^2+^ handling enzymes, proteins, channels and transporters located in the plasma membrane and in Ca^2+^ storage organelles, which work in concert to fine tune a temporally and spatially precise Ca^2+^ signal^53,54^. The sarcoplasmic reticulum (SR) plays an important role in orchestrating the movement of Ca^2+^ during each contraction and relaxation. Excitation leads to the opening of voltage gated L-type Ca^2+^ channels, allowing the entry of a small amount of Ca^2+^ into the cell^54,55^. Through a coupling mechanism between the L-type Ca^2+^ channel (LTCC) and the SR Ca^2+^ release channel (ryanodine receptor 2 – RyR2), a larger amount of Ca^2+^ is released through a process termed Ca^2+^-induced Ca^2+^ release, activating the myofilaments, leading to contraction^54,55^. During relaxation, Ca^2+^ is re-accumulated back into the SR by the SR Ca^2+^-ATPase pump (Serca2a) and extruded extracellularly primarily by the sarcolemmal Na^+^/Ca^2+^ exchanger-1. The plasma membrane Ca^2+^-ATPase pump and the mitochondrial uniporter may also contribute to this process, albeit minimally^54,55^. The contribution of each of these mechanisms for lowering cytosolic Ca^2+^ varies with species, with more than 90% of the removal is attributed to Serca2a in rodents^55^. We demonstrated in this study that clearance of intracellular Ca^2+^ through pharmacological or genetic activation of Serca2a significantly attenuated the activation of NFATc and resistin. Although we focused in this study on the contribution of Serca2a to calcium removal given its predominant Ca^2+^ removal in rodents and its well documented critical role in heart failure^31,56^, it would be interesting to examine in future studies the potential contribution, if any, of other Ca^2+^ handling molecules to resistin regulation.

In summary, diabetic hearts are characterized by elevated resistin’s mRNA and protein levels. We provide the first evidence of a Ca^2+^-sensitive mechanism underlying resistin transactivation. Normalization of diabetes-associated Ca^2+^ dyshomeostasis through myocardial-specific restoration of Serca2a expression in diabetic hearts controls resistin transcriptional activity via manipulation of NFATc. Our current findings lend further support of Serca2a as potential therapy for hyper-resistinemia conditions in addition to its broader metabolic, mechanical and energetic benefits in heart failure^31,56^. Given the deleterious effects of aberrant resistin expression in diabetic hearts^27–29,37^, measures to lower and normalize its levels may constitute a reasonable mechanism to mitigate diabetes-induced cardiomyopathy.

## Methods

### Animals, Viral Injection and Small Molecule Treatment

Male 8 to 10-week old *ob/ob* mice (B6.Cg-Lep^ob^/J; 000632) and lean *ob/*+mice (C57BL/6J; 000664) were obtained from Jackson Laboratory (n = 10/group). Mice were divided into 6 groups: lean and o*b/ob* treated with either vehicle (10% DMSO, 10% Tween 80 in 0.9% NaCl) or CDN1163 (50 mg/kg), intraperitoneally 3×/week for 30 days (pharmacology protocol); lean and o*b/ob* injected with AAV9.Empty or AAV9.Serca2a (3 × 10^12^ viral particles) via tail-vein for 12 weeks (gene therapy protocol). Male 8–10-week old Serca2a^−/−^ mice (*n* = 10) were generated as described previously^57^. Animals were handled as approved by the Mount Sinai Institutional Animal Care and Use Committee in accordance with the Principles of Laboratory Animal Care by the National Society for Medical research and the Guide for the Care and Use of Laboratory Animals (National Institutes of Health Publication No. 86-23, revised 1996).

### Cell Culture and Treatment

Rat cardiac myocyte cells (H9c2) were grown in DMEM supplemented with 10% FBS and 1X cocktail of pen/strep antibiotics. Cells were either infected with Ad.βgal, Ad.Serca2a (at different multiplicity of infection as indicated) and Ad.NFATc or were exposed to CDN1163 (10 μM), 1,2-Bis(2-aminophenoxy)ethane-N,N,N′,N′-tetra-acetic acid tetrakis(acetoxymethyl ester)/BAPTA-AM (2 μM), or Ca^2+^ (4 mM) in low glucose (5 mM) or high glucose (25 mM) to mimic diabetic condition for the indicated times (20 mM mannitol is added to verify glucose induced osmotic effects). Cells were lysed and harvested for real-time-PCR and western analysis.

### Western Blotting

Cardiomyocytes and isolated heart tissues were homogenized in lysis buffer (Cell Signaling Tech.) containing protease and phosphatase inhibitors. Fractionated cytoplasmic and nuclear protein lysates (20–40 μg) were separated and applied to SDS-PAGE and transferred onto PVDF membrane (BioRad). Antibodies used were phospho- or total against AMPKα, phospho-AMPKα-T172, (Cell Signaling Technology), histone 3 (H3) (Genetex), NFATc and β-actin (Santa Cruz Biotechnology) and resistin (Millipore). Serca2a antibody is custom made in our lab). β-actin expression verified cytosolic protein loading while H3 served as nuclear specific internal control.

### Constructs and Luciferase Assay

The NFATc over-expression plasmid pEGFP-C1 NFATc was obtained from Addgene^44^. Adenoviruses encoding Serca2a and VIVIT, an NFATc inhibitor, were constructed as previously described^58^. The resistin promoter (−1000 to −1 bp) was amplified from mouse genomic DNA and cloned into the pGL3-luciferase reporter plasmid yielding pRetnP-GL3. The cloned resistin promoter construct was confirmed by sequencing. H9c2 cells were co-transfected with NFATc-plasmid, pRetnP-GL3 plasmid or empty vector using lipofectamine 2000 reagent (Invitrogen, Thermo Scientific). Twenty four hours later cells were treated with CDN1163 or Ca^2+^ and infected with either Ad.Serca2a or Ad.VIVIT and incubated for an additional 24 hours. The luciferase activity was measured using Promega luciferase assay.

### ChIP Assay

H9c2 cells were treated with Ca^2+^ (4 mM)^34^ for 30 minutes. The crosslinking of DNA-nuclear factor proteins complex was carried out using 1% formaldehyde for 10 min at room temperature. The crosslinking was quenched by addition of 0.125 M glycine to the media and incubated with shaking for 5 min at room temperature. Cells were washed two times with ice-cold PBS and scraped into PBS containing protease inhibitors. The cell suspension was centrifuged and the pellet was resuspended in ChIP buffer (50 mM HEPES-KOH [pH 7.5], 140 mM NaCl, 1 mM EDTA [pH 8], 1% Triton X-100, 0.1% sodium deoxycholate, 0.1% SDS, and protease inhibitors) and followed by incubation for 10 minutes on ice. The lysate was sonicated to shear DNA to an average fragment size of 200–1000 bp and centrifuged to remove cell debris. The isolated lysate was diluted in RIPA buffer containing protease inhibitors. To immuno-precipitate chromatin, appropriate amounts of ChIP-grade NFATc antibody (sc-13036, Santa Cruz Biotech.) or isotype-control IgG were added overnight at 4 °C with rotation. This step was followed by addition of ChIP-grade protein A/G PLUS-agarose (sc-2003, Santa Cruz Biotech.) beads and incubated for 2 hours at 4 °C with rotation. The immunoprecipitated samples were centrifuged to remove supernatant. The pellets were washed once in low salt wash buffer (0.1% SDS, 1% Triton X-100, 2 mM EDTA, 22 mM Tris-HCl [pH 8], 150 mM NaCl), once in high salt wash buffer (0.1% SDS, 1% Triton X-100, 2 mM EDTA, 20 mM Tris-HCl [pH 8], 500 mM NaCl), and once in LiCl wash buffer (0.25 M LiCl, 0.1% NP-40, 1% sodium deoxycholate, 1 mM EDTA, 10 mM Tris-HCl [pH 8]). The chromatin bound to protein A/G PLUS-agarose beads was then eluted from beads with 200 μl elution buffer (100 mM NaHCO3 and 1% SDS). After RNase and proteinase K digestion, DNA was isolated using QIAquick PCR purification kit (Qiagen) and analyzed by PCR to determine the binding of NFATc to resistin gene promoter. The region of the mouse resistin promoter from − 1 to −2500 bp was taken to identify putative NFATc binding sites (consensus sequence: (T/A)GGAAAA(A/N)(A/T/C) by using online transcription factors binding prediction software PROMO 3.0; http://alggen.lsi.upc.es) and Genomatix MatInspector. The following primers were used for rat resistin promoter (forward 5′-TTTGTCCAAATGAGGCT TCC-3′, reverse 5′-GGTCTGCCATAGCCTCTCAG-3′. The PCR amplification was performed using CloneAmp HiFi PCR premix (Cat. No. 639298, ClonTech).

### Real-Time PCR

Total RNA was isolated from mice hearts or cultured cells using TRIzol and complementary DNA (cDNA) was generated using a High Capacity cDNA Reverse Transcription Kit (Applied Biosystems) according to the manufacturer’s instructions. Real-time PCR was performed with the Perfecta SYBR Green FastMix, Low ROX (Catalogue No. 95074-012, Quanta Biosciences) in a 7500 Real-time PCR (Applied Biosystems) after adjusting the threshold cycle (Ct). Reactions were performed in triplicates with 18S internal as control. Relative quantification of mRNA levels were analyzed by the ΔΔCT method and expressed as fold increase relative to the control. The following primers were used; rat resistin: forward 5′-CCAGCTGCAATGAAGAACAC-3′ and reverse 5′ CCGCTGTCCAGTCTATGCTT; mouse resistin: forward 5′-TCATTTCCCCTCCTTTTCCTTT-3′ and reverse 5′-TGGGACACAGTGGCAT GCT-3′. Mouse/rat NFATc: forward 5′-TACAGCAACAAGCGGGTGTC-3′ and reverse 5′ CGGA GAGATGAGTCTGGTAGGG-3′. 18S control, forward 5′-AGTCCCTGCCCTTTGTACACA-3′ and reverse 5′-CGATCCGAGGGCCTCACTA-3′.

### Statistics

Data are expressed as the means ± S.E. The significance of the differences in mean values was evaluated by using unpaired Student’s *t* test or non-parametric one way analysis of variance (ANOVA) with Mann-Whitney post-hoc test where appropriate from at least three independent experiments in triplicates. Values of *p* < 0.05 were considered to be statistically significant.

### Prior Presentation

Parts of this study were presented in abstract form at the Annual Meeting of the American Heart Association 2017, Anaheim CA, 11–15 November 2017.
